# Investigation of the relationship between venous thromboembolism and thrombophilic variants

**DOI:** 10.1590/1806-9282.20241171

**Published:** 2025-03-17

**Authors:** Ayca Kocaaga, Müfide Okay Özgeyik

**Affiliations:** 1 Eskisehir City Hospital, Department of Medical Genetics – Eskişehir, Turkey.; 2 Eskisehir City Hospital, Department of Hematology – Eskişehir, Turkey.

**Keywords:** Deep vein thrombosis, Plasminogen activator inhibitor 1, Factor XIII, Pulmonary embolism, Venous thromboembolism

## Abstract

**OBJECTIVE:**

Venous thromboembolism could be manifested as deep venous thrombosis or pulmonary embolism. The aim of this study was to assess the impact of genetic risk factors including prothrombin 20210, Factor V Leiden, plasminogen activator inhibitor 4G/5G, and Factor XIII V34L on the occurrence of venous thromboembolism in patients.

**METHODS:**

This study was conducted on 128 patients with deep venous thrombosis and 84 patients with pulmonary embolism. The diagnosis of venous thromboembolism was based on the patient's history, clinical findings, and D-dimer and confirmed by Doppler ultrasonography or computed tomography angiography. After confirmation of venous thromboembolism diagnosis, both groups were assessed for the four abovementioned mutations.

**RESULTS:**

The majority of deep venous thrombosis patients were much younger than pulmonary embolism patients, with a median age of 51.7 years. It was observed that plasminogen activator inhibitor 4G/5G was most commonly represented in the deep venous thrombosis (44.5%) group, followed by the pulmonary embolism (44.0%) group. The second-highest frequency of Factor XIII V34L was observed in the deep venous thrombosis (28.1%) and pulmonary embolism (32.1%) groups. Factor V Leiden heterozygosity was also common in the deep venous thrombosis (18.0%) and pulmonary embolism (27.4%) groups. We found that coagulation factor II (FII) G20210A heterozygosity was the least in the deep venous thrombosis (10.9%) and pulmonary embolism (9.5%) groups.

**CONCLUSION:**

To date, only a few studies have been thrombophilia parameters associated with venous thromboembolism, particularly Factor XIII V34L, in Turkish population with venous thromboembolism patients. Our findings suggest that genetic risk factors play a role in the formation of venous thromboembolism.

## INTRODUCTION

Venous thromboembolism (VTE), including deep venous thrombosis (DVT) and pulmonary embolism (PE), is the third most common acute cardiovascular disorder and has high morbidity and mortality rates^1^. Susceptibility in thrombophilia, like in all multifactorial diseases, implicates the interaction between environmental factors, such as diet or smoking, as well as genetic factors^2^. The heritability of VTE has been estimated at about 60%. Given the critical role of genetic factors in the pathogenesis of the disease, single-nucleotide polymorphisms (SNPs) have now become the research topic of many researchers^2,3^.

Factor V (FV) is a 330 kDa protein that plays a key role in blood coagulation, converts prothrombin to thrombin, and is inactivated by protein C (PC), thereby modulating the amount of thrombin produced^4^. A widely studied SNP in the FV gene is the G1691A mutation, known as FV-Leiden (FVL) (rs6025)^5^. FVL is probably the most important hereditary thrombosis-associated factor in Caucasians, with heterozygotes exhibiting up to a 10-fold greater relative risk and Leiden homozygotes exhibiting a 50-100-fold greater relative risk of venous thrombosis^5,6^.

Coagulation factor II (FII) is a plasma glycoprotein that is activated to thrombin by coagulation factors like FXa and FVa. The gene encoding FII or prothrombin contains another common defect in its 3′ untranslated region (G20210A)^7^. The heterozygous and homozygous types of the prothrombin G20210A gene mutation have a 3-8-fold higher risk and an 18-80-fold higher risk of thromboembolic events, respectively^8^.

Plasminogen activator inhibitor-1 (PAI-1) (SERPINE1) 4G/5G gene polymorphism has been identified as a thrombophilic factor in recent studies^9^. The 4G/5G variant involves a single-nucleotide deletion/addition mutation in the guanine position 675 of the promoter region^10^. This variant causes two different alleles, 4G and 5G, which alter the regulation of the PAI-1 protein. Individuals with the wild-type (5G/5G) genotype have higher concentrations of the PAI-1 protein^9^. Many studies to date have found that the 4G/4G genotype is associated with a higher risk of thrombosis^9,10^.

Factor XIII (FXIII) is a 320 kDa multifunctional glycoprotein involved in coagulation, fibrinolysis, and inflammation in a heterotetramer structure consisting of 2A and 2B subunits. The V34L (Val34Leu) polymorphism is located at exon 2, resulting in a faster activation of FXIII by thrombin^11,12^. This rare FXIII 34Leu variant increases the resistance to clot formation and could decrease thrombosis risk^13^. The relationship between the V34L polymorphism of FXIII and the risk of VTE and PE is still controversial^11-13^.

In this study, we aimed to investigate the association between four genetic variants, including FVL G1691A, prothrombin G20210A, PAI-1 4G/5G, and FXIII V34L, and the risk of VTE in adults from Turkey.

## METHODS

This retrospective study was conducted on patients who were diagnosed with VTE in a tertiary care hospital between May 2022 and May 2023. The study was approved by the Ethics Committee of (anonymized) (ESH/GOEK-2023/4). The demographic data of patients, such as age and gender, VTE and PE development patterns, and comorbidities, were recorded. Informed consent was obtained from each participant.

Blood samples were collected in tubes containing ethylenediaminetetraacetic acid (EDTA) for DNA isolation. Genomic DNA was isolated from individuals by using the QIAamp DNA Blood Mini Kit (Qiagen Inc., Germany). To determine the prevalence of the mutations in the thrombophilia panel, the subjects were genotyped for FV G1691A, FII G20210A, PAI 4G/5G, and FXIII V35L variants using real-time polymerase chain reaction with Cobas Z 480 LightCycler (Roche Molecular Diagnostics, CA, USA) according to the guidelines of the manufacturer. Factor V Leiden Kit (Roche Molecular Systems, Branchburg, New Jersey, USA) and Factor II (prothrombin) G20210A Kit (Roche Molecular Systems, Branchburg, New Jersey, USA) were used for genotyping FV G1691A and FII G20210A mutations, respectively. LightCycler FastStart DNA Master containing speciﬁc primers and probes was used for genotyping PAI 4G/5G and FXIII V35L mutations, respectively. We investigated the frequency of thrombophilic variants (FV G1691A and FII G20210A mutations and PAI 4G/5G and FXIII V35L polymorphisms) calculated for this retrospective study.

Statistical analysis was performed with the SPSS 23.0 software. Data were expressed as mean or frequency. The t-test, chi-square test, or Fisher's exact test was used to evaluate the statistical difference. The odds ratios (OR) and 95% confidence intervals were also calculated. p<0.05 was considered statistically significant. The gene counting method was used for allele frequency calculations.

## RESULTS

The general characteristics of 212 patients are listed in Table 1. The mean ages of patients in the DVT and PE groups were 43.406 and 51.738 years, respectively. There was a significant difference between the patient groups in terms of mean age. It has been observed that patients with DVT are younger compared to patients with PE (Table 1; p<0.001). Of the 212 patients with VTE, 128 (60.0%) had isolated DVT, and 84 (40.0%) had PE. There was no significant difference in the frequency of the traditional risk factors of cardiovascular diseases (obesity, major surgeries, family history, and cancer) between the patient groups. The observed genotype distribution of thrombotic genes was in agreement with the Hardy-Weinberg equilibrium in both patient groups (VTE and PE) (p>0.05). Of the 212 patients, 199 (93.8%) had one or more mutations. Three or more than three mutations were detected in 10 patients (4.7%). PAI 4G/5G was most commonly represented in the DVT (44.5%) group, followed by the PE (44.0%) group. The frequency of the homozygous 4G/4G genotype was 25.0 and 21.4% in the DVT and PE groups, respectively. The second highest frequency of FXIII V34L (G>T) was observed in the DVT (28.1%) and PE (32.1%) groups, respectively. The frequency of the homozygous FXIII V34L genotype was 2.3 and 1.2% in the DVT and PE groups, respectively (Table 2). There were 34 (16.0%) patients with PAI 4G/5G and FXIII V34L digenic heterozygosity, of which 20 had DVT (15.6%) and 14 (16.6%) had PE. FVL heterozygosity was common in the DVT (18.0%) and PE groups (27.4%). The frequencies of homozygous mutations for FVL were 4.7 and 2.4% in the DVT and PE groups, respectively. We found that the prevalence of FII G20210A heterozygosity was the least in the DVT (10.9%) and PE (9.5%) groups. Moreover, the homozygous genotype for FII G20210A was present in only one patient in the DVT (0.8%) group (Table 2). A statistically significant relationship was not found between the groups in terms of allele frequencies (p>0.05, Table 3).

**Table 1:** Clinical characteristics of the study groups.

Characteristics			DVT n=128 (60%)	PE n=84 (40%)	p-value
Mean age±SD			43.406±13.045	51.738±15.632	**<0.001** * ɵ
Female			59 (46.1%)	48 (57.1%)	0.116ɸ
Male			69 (53.9%)	36 (42.9%)	
Risk factors					
	Family history	Yes	25 (19.5%)	20 (23.8%)	0.456ɸ
		No	103 (80.5%)	64 (76.2%)	
	Major surgeries	Yes	5 (3.9%)	6 (7.1%)	0.350ɸ
		No	123 (96.1%)	78 (92.9%)	
	Obesity	Yes	6 (4.7%)	4 (4.8%)	1.000ɸ
		No	122 (95.3%)	80 (95.2%)	
	Cancer	Yes	6 (4.7%)	9 (10.7%)	0.094ɸ
		No	122 (95.3%)	75 (89.3%)	

* p<0.001;

ɵ independent sample t-test;

ɸ chi-square test;

SD: standard deviation; DVT: deep venous thrombosis; PE: pulmonary embolism; statistically significant values are denoted in bold.

**Table 2:** Genotype frequencies of Factor V Leiden G1691A, prothrombin G20210A, plasminogen activator inhibitor 4G/5G, and Factor XIII (V35L).

Genotypes		DVT n=128 (60%)	PE n=84 (40%)	Total	p-value
FVL (G>A)					
	Wild (GG)	99 (77.3%)	59 (70.2%)	158 (74.5%)	0.521ɸ
	Heterozygous (GA)	23 (18.0%)	23 (27.4%)	46 (21.6%)	
	Homozygous (AA)	6 (4.7%)	2 (2.4%)	8 (3.9%)	
FII 20210 (G>A)					
	Wild (GG)	113 (88.3%)	76 (90.5%)	189 (89.1%)	0.523ɸ
	Heterozygous (GA)	14 (10.9%)	8 (9.5%)	22 (10.3%)	
	Homozygous (AA)	1 (0.8%)	0 (0%)	1 (0.6%)	
FXIII (V34L; G>T)					
	Wild (GG)	89 (69.5%)	56 (66.7%)	145 (68.3%)	0.812ɸ
	Heterozygous (GT)	36 (28.1%)	27 (32.1%)	63 (29.7%)	
	Homozygous (TT)	3 (2.3%)	1 (1.2%)	4 (6%)	
PAI 4G/5G					
	Wild (5G/5G)	39 (30.5%)	29 (34.5%)	68 (32.0%)	0.765ɸ
	Heterozygous (5G/4G)	57 (44.5%)	37 (44.0%)	91 (42.9%)	
	Homozygous (4G/4G)	32 (25.0%)	18 (21.4%)	50 (25.1%)	

ɸ Chi-square test;

DVT: deep venous thrombosis; PE: pulmonary embolism; FVL: FV Leiden; FXIII: Factor XIII; FII: coagulation factor II.

**Table 3:** Allele frequencies of Factor V Leiden G1691A, prothrombin G20210A, plasminogen activator inhibitor 4G/5G, and Factor XIII (V35L).

Allele frequencies		DVT n=128 (60%)	PE n=84 (40%)	Total	p-value
FVL (G>A)					
	G (wild)	221 (86.3%)	141 (83.9%)	362 (85.4%)	0.438ɸ
	A (mutant)	35 (13.7%)	27 (16.1%)	62 (14.6%)	
FII 20210 (G>A)					
	G (wild)	240 (93.7%)	160 (95.2%)	400 (94.3%)	0.532ɸ
	A (mutant)	16 (6.3%)	8 (4.8%)	24 (5.7%)	
FXIII (V34L; G>T)					
	G (wild)	214 (83.5%)	139 (82.7%)	353 (83.2%)	0.359ɸ
	T (mutant)	42 (16.5%)	29 (17.3%)	71 (16.8%)	
PAI 4G/5G					
	5G (wild)	135 (52.7%)	95 (56.5%)	230 (54.3%)	0.584ɸ
	4G (mutant)	121 (47.3%)	73 (43.5%)	194 (45.7%)	

ɸ Chi-square;

DVT: deep venous thrombosis; PE: pulmonary embolism; FVL: FV Leiden; PAI: plasminogen activator inhibitor; FXIII: Factor XIII; FII: coagulation factor II.

## DISCUSSION

The evaluation of genetic risk factors has become an integral part of the diagnostic evaluation of patients presenting with the signs and symptoms of VTE. In various studies, FII G20210A, FVL G1691A, MTHFR C677T, and PAI 4G/5G polymorphism regions have been particularly reported to be risk factors for venous thrombosis. In our study groups, the highest (mutant) allele frequencies were detected in the PAI 4G/5G and FXIII (V34L; G>T) polymorphisms (Table 3). In a study on the Croatian population, the FVL G1691A mutation was identified to be the most common and the FII gene G20210A was identified to be the second most common mutation^14^.

The prevalence of FVL varies in different populations, and the differences in its distribution can be explained by ethnic origin and geographical differences. Its prevalence is around 3-5% (occasionally up to 15% in certain areas) in European countries, 14% in Greece, and between 7.1 and 11.7% in Turkey^14-16^. In our study, the prevalence of FVL was 22.7 and 29.8% in the DVT and PE groups, respectively (Table 2), and it was above the threshold value in many normal populations, including Turkey^4-6,15^.

In the literature, the FVL mutation was in the heterozygote state in the majority of VTE patients^4-6,16^. However, homozygous mutations were also evident although in a few. It has been identified in our trial that 54 (25.5%) of the 212 cases with thrombosis had the FVL mutation (Table 2). Heterozygosity was evident in 46 (21.6%) cases, of which 8 (3.9%) had a homozygous mutation. Gül et al. showed that the prevalence of the FVL mutation in the heterozygous state was found to be 37.5% in patients with DVT^17^. Akar et al. reported that the prevalence of the FVL mutation in thrombosis cases was 9.8%^18^. The FVL mutation prevalence of 14.6% found in our VTE group suggests a strong concomitance of the FVL mutation especially with VTE.

FII G20210A mutation, a risk factor for hereditary thrombophilia, is associated with a 7-10-fold increased lifetime risk of venous or arterial thrombosis^5^. In Western countries, the FIIG20210A mutation has been found in 6-8% of patients with venous thrombosis^6^. Akar et al. reported the frequency of the FII20210A mutation in the Turkish population as 2.7%^19^. In another Turkish study, the frequency of the FII20210A mutation was found to be 4.9% in cases with DVT^20^. In the present study group, the prevalence of PT20210A mutations was 10.9% (Table 2), which is higher than the reported frequencies of healthy Turkish adults in various studies (6.3, 4.9, and 2.7%)^19,20^.

The 4G/5G insertion/deletion polymorphism in the promoter region (-675) of the PAI-1 gene located on chromosome 7 is one of the most frequently studied polymorphisms of the gene^9^. There are only very few studies reported from Turkey regarding this gene polymorphism. Oguzulgen et al. found the 4G/5G polymorphism rate to be 44.1% in PE patients and 55.9% in the control group. In their study, the genotype frequencies of PAI 5G/5G, 5G/4G, and 4G/4G were determined as 30.8, 44.1, and 25.2%, respectively. They found no association between the PAI-1 polymorphism and PE^20^. In a study conducted on VTE patients from Turkey, the genotype frequencies of PAI 5G/5G, 5G/4G, and 4G/4G were found to be 24.8, 50.4, and 24.8%, respectively^21^. In our patient population (DVT+PE), the frequencies of PAI genotypes were 32.0% (5G/5G), 42.9% (5G/4G), and 25.1% (4G/4G) (Table 2). Our results are similar to the literature results.

Several case-control studies have reported that the FXIII Val34Leu phenotype is resistant to coronary artery disease and stroke^12^. Renner et al. reported the frequency of the 2 FXIII alleles in patients with VTE. In 154 patients, the prevalence of heterozygous form was found to be 31.8% and that of homozygous Leu34 was found to be 5.2%^22^. Another case-control study of 97 patients with DVT and an equal number of healthy control subjects found no significant association of the Val34Leu polymorphism with thrombosis^23^. The Val34Leu polymorphism was found in 39 (30.5%) of 128 DVT patients, compared to 28 (33.3%) of 84 PE patients (Table 2). There was no study reporting FXIII genotype and allele frequencies in VTE patients from Turkey. Therefore, this study is the first to report the genotype frequencies of the FXIII polymorphism in DVT and/or PE in the Turkish population. In our study, the rate of the FXIII V34L heterozygous mutation was 29.7% in VTE (DVT+PE) patients, of which 6% were homozygous.

## CONCLUSION

In summary, we recommend probing for predisposing genetic risk factors in patients with thromboembolic complications, such as DVT and PE. In recent years, many gene mutations have been found to cause venous thromboembolic disease. The common causes of VTE in the general population should be well known and appropriate precautions should be taken. As a result, every patient should be evaluated for hereditary thrombophilia in the diagnosis and treatment planning of VTE.
